# Estimation of high risk pregnancy contributing to perinatal morbidity and mortality from a birth population-based regional survey in 2010 in China

**DOI:** 10.1186/1471-2393-14-338

**Published:** 2014-09-30

**Authors:** Libo Sun, Hongni Yue, Bo Sun, Liangrong Han, Zhaofang Tian, Meihua Qi, Shuyan Lu, Chunming Shan, Jianxin Luo, Yujing Fan, Shouzhong Li, Maotian Dong, Xiaofeng Zuo, Yixing Zhang, Wenlong Lin, Jinzhong Xu, Yongbo Heng, Huai’an Perinatal-Neonatal Study Group

**Affiliations:** Departments of Pediatrics and Neonatology, Children’s Hospital and the Institutes of Biomedical Science, Fudan University, and the Laboratory of Neonatal Medicine, National Health and Family Planning Commission, Shanghai, 201102 China; Departments of Neonatology and Obstetrics, Huai’an Women and Children’s Hospital, Huai’an, Jiangsu 223002 China; Departments of Neonatology and Obstetrics, Huai’an First People’s Hospital, Huai’an, Jiangsu 223002 China; Departments of Pediatrics and Obstetrics, Huai’an Second People’s Hospital, Huai’an, Jiangsu 223002 China; Departments of Pediatrics and Obstetrics, Huaiyin District Hospital, Huai’an, Jiangsu 223300 China; Departments of Pediatrics and Obstetrics, Chuzhou District Hospital, Huai’an, Jiangsu 223200 China; Departments of Pediatrics and Obstetrics, Lianshui County Hospital, Huai’an, Jiangsu 223400 China; Departments of Pediatrics and Obstetrics, Xuyi County Hospital, Huai’an, Jiangsu 211700 China; Departments of Pediatrics and Obstetrics, Xuyi County Hospital for Traditional Chinese Medicine, Huai’an, Jiangsu 211700 China; Departments of Pediatrics and Obstetrics, Hongze County Hospital, Huai’an, Jiangsu 223100 China; Departments of Pediatrics and Obstetrics, Jinhu County Hospital, Huai’an, Jiangsu 211600 China

**Keywords:** Morbidity, Mortality, Perinatology, Perinatal risks, Neonatal outcomes, High-risk pregnancy

## Abstract

**Background:**

Neonatal mortality reduction in China over past two decades was reported from nationwide sampling surveys, however, how high risk pregnancy affected neonatal outcome is unknown. The objective of this study was to explore relations of pregnancy complications and neonatal outcomes from a regional birth population.

**Methods:**

In a prospective, cross-sectional survey of complete birth population-based data file from 151 level I-III hospitals in Huai’an region in 2010, pregnancy complications were analyzed for perinatal morbidity and mortality in association with maternal and perinatal characteristics, hospital levels, mode of delivery, newborn birth weight and gestational age, using international definition for birth registry and morbidities.

**Results:**

Pregnancy complications were found in 10% of all births, in which more than 70% were delivered at level II and III hospitals associated with higher proportions of fetal and neonatal death, preterm birth, death at delivery and congenital anomalies. High Cesarean section delivery was associated with higher pregnancy complications, and more neonatal critical illnesses. The pregnancy complications related perinatal morbidity and mortality in level III were 2–4 times as high as in level I and II hospitals. By uni- and multi-variate regression analysis, impact of pregnancy complications was along with congenital anomalies and preterm birth, and maternal child-bearing age and school education years contributing to the prevalence.

**Conclusions:**

This survey revealed variable links of pregnancy complications to perinatal outcome in association with very high Cesarean section deliveries, which warrants investigation for causal relations between high risk pregnancy and neonatal outcome in this emerging region.

## Background

In the past decade there has been a dramatic decline in mortality of children under 5 years old in China, from 60/1,000 in early 1990 to a level below 15/1,000 live births in 2012 as recently reported by the National Health and Family Planning Commission (http://www.nhfpc.gov.cn, 2013 July), in great extent associated with the reduction of neonatal mortality [1–8], i.e., from 40/1,000 to <7/1,000 live births, along with significantly reduced maternal death rate (<25/100,000 births) and narrowed disparities between rural and urban death rates. This was mainly due to effective implementation of prenatal care from early pregnancy, hospital delivery, delivery room resuscitation and neonatal special care, and medications for infectious diseases, diarrhea and malnutrition, among others [7–13]. Although national vital statistics revealed this trend, it is not known yet on how the high risk pregnancy would link to perinatal and neonatal death rate in total births and live births. As there are great variations in different regions in China, and perinatal and neonatal mortality contributed to the under five children’s mortality by 60-70%, how to improve the perinatal and neonatal survival is a big challenge not only for the health care givers and clinical technology, but also public health care administrators and practitioners, policy makers, and child welfare, special care and education professionals in the whole society and local communities. A variety of questions are raised from potential burden in technologies and resource to ethics regarding limit of viability, where lacking of systematic information and reporting, diagnostic means and care standard, resources of public health care system, and staff competence and availability at community level, etc., are commonly encountered but compelling to find solutions for sustainability in the current development in developing countries.

There is no complete birth-population-based regional or provincial data of survey available so far regarding high risk pregnancy contributing to the perinatal and neonatal morbidity and mortality. Existing information come mostly from surveys based on the approaches by sampling of selected level II hospitals of rural counties and urban districts for total births and related death causes, which provided estimated nation-wide data [7, 8], but may not provide perinatally associated maternal-fetal and neonatal disease incidence and death rate from different hospital level based information, neither is valid for estimation of nation-wide perinatal and neonatal morbidity and mortality appropriate to the need of country’s development. Besides, in the nation-wide reporting system for regional vital statistics, the births earlier than 29 weeks of gestational age (GA) are not included whereas advanced neonatal special care at regional and county/city hospitals is common now in many emerging regions for neonates born before 29 weeks of GA or <1,000 g of birth weight (BW) to survive. Therefore, it does not provide perinatal and neonatal information associated with pregnant status and complications, or incidence, management and outcome (death and complications) of specific maternal-fetal and neonatal disorders. Neither does it provide information of how many deaths are related to inappropriate coverage of prenatal care in regions with different levels of health care service. Thus, listing of neonatal death rate and their causes only has shown a trend of changes in neonatal death and underlying causes, but lacks comprehensive view as to what extent the obstetric and neonatal clinic performance as well as regional public health policy and resource really contribute to the overall and specific neonatal outcome. Considering the high birth population and dramatic improvement of perinatal and neonatal care in many emerging regions of the country, an in-depth study of high risk pregnancy and prenatal morbidity related fetal and neonatal outcome is compelling. In this regard, we prospectively conducted a regional complete birth population-based, cross-sectional survey in combination with most, if not all, of the hospitalized high risk pregnancies and neonates with various diseases, which provides information including high risk pregnancy contributing to the perinatal-neonatal morbidity and mortality using epidemiological methodology in Huai’an [14], Jiangsu province, China.

The objective of this analysis from the cross-sectional survey-based descriptive data was to delineate high risk pregnancy linking to the fetal and neonatal morbidity and mortality. Characterizing the incidence of prenatal and intrapartum complications, mode of delivery associated perinatal and neonatal risks and outcomes from regional hospitals may facilitate an estimation of nation-wide pregnancy and childbirth related efficacy of policies, strategies and programs as well as burden and resource limitation, for understanding the trend and perspectives of woman and child health care development in China.

## Methods

### Summary of study region and population

The survey was conducted in Huai’an city, a prefectural region, Jiangsu province, located in the eastern part of China with a population of 5,400,000 and nearly 60,000 births in 2009, with approximately 5% migrant people for seasonal, economic living in this region. There were almost equal proportion of residents from both urban and rural origin, and economic levels, judged by gross domestic production per head, were similar to that of the national averages in 2010. Its socio-cultural tradition was also representative of average in most of the east and midland provinces of the country. We assumed that a cross-sectional, perinatal-neonatal survey in Huai’an region should represent the situation in most emerging regions of China, with intermediate development in both economics and woman and child health care, presumably accounting for up to 50% of the total Chinese population (1.34 billion in 2010 census), as characterized by high hospital delivery (>99%), health insurance for most rural residents (>95%) and health care infrastructure for coverage and availability.

### Synopsis of the study protocol

Details of the collaborative study group for perinatal-neonatal care in Huai’an region, data collection and quality control was reported elsewhere [14], and are summarized below:

Study setting: 151 level I-III hospitals (Table 1) providing child delivery and neonatal care service (level II-III hospitals).

**Table 1:** **Comparison of all child births and related morbidity and mortality in different levels of hospitals in Huai’an**

Hospital level	I	II	III	P
Number of hospitals	129	15	7	
All births	31,680 (52.3)	19,767 (32.6)	9,168 (15.1)	
Fetal deaths/stillbirth	36 (0.1)	143 (0.7)	71 (0.8)	
Live births	31,644 (99.9)	19,624 (99.3)	9,097 (99.2)	
Males	16,900 (53.5)	10,539 (53.5)	4,967 (55.1)	0.018
Gestational age (weeks)	39.9±1.2	39.6±1.5	39.0±2.1	<0.001
Birthweight (grams)	3,469±447	3,443±495	3,338±604	<0.001
Preterm births	404 (1.3)	776 (3.9)	1,059 (11.6)	<0.001
Low birthweight	395 (1.2)	545 (2.8)	751 (8.3)	<0.001
Multiple-births	300 (0.9)	385 (1.9)	403 (4.4)	<0.001
Congenital anomalies	117 (0.4)	119 (0.6)	78 (0.9)	<0.001
Cesarean section	15,566 (49.2)	11,155 (56.6)	5,243 (57.5)	<0.001
Pregnancy complications	1,766 (5.6)	2,380 (12.1)	1,905 (21.1)	<0.001
Hypertension	327 (1.0)	410 (2.1)	279 (3.0)	<0.001
PROM	1,062 (3.3)	1,211 (6.1)	1,017 (11.1)	<0.001
Anemia	236 (0.7)	363 (1.8)	147 (1.6)	<0.001
Maternal age (years)	25.5±5.2	26.1±5.1	26.7±4.9	<0.001
Delayed childbearing	2,543 (8.1)	1,698 (8.6)	824 (9.0)	0.006
>9 years’ education	2,223 (7.4)	4,261 (23.2)	3,855 (50.0)	<0.001
Amniotic fluid volume				<0.001
Normal	29,162 (93.4)	18,037 (92.2)	7,665 (90.6)	
Polyhydramnios	192 (0.6)	134 (0.7)	150 (1.8)	
Oligohydramnios	1,873 (6.0)	1,400 (7.2)	647 (7.6)	
Amniotic contamination				<0.001
Normal	27,647 (87.5)	16,240 (82.5)	7,639 (86.8)	
Grade I	1,845 (5.8)	1,105 (5.6)	286 (3.2)	
Grade II	1,356 (4.3)	1,069 (5.4)	324 (3.7)	
Grade III	732 (2.3)	1260 (6.4)	548 (6.2)	
Apgar score				
1-min ≤7	783 (2.5)	767 (3.9)	740 (8.2)	<0.001
1-min ≤3	75 (0.2)	176 (0.9)	98 (1.1)	<0.001
5-min ≤7	105 (0.3)	193 (1.0)	244 (2.7)	<0.001
5-min ≤3	35 (0.1)	137 (0.7)	64 (0.7)	<0.001
Deaths^a^	56 (0.2)	156 (0.8)	90 (1.0)	<0.001

All values are given in numbers and percentage (% of all births in each hospital level category) or means±SD. Definition of abbreviations: PROM, premature rupture of membrane.

^a^Including fetal deaths/stillbirth and neonatal deaths immediately at delivery but counted as live births.

Study design: prospective, cross-sectional survey of complete birth data from hospital deliveries including pregnancy complications and perinatal morbidities.

Study population: all births including fetal death, stillbirth and live births collected from Jan 1 to Dec 31, 2010.

Sample size: based on 2009 total births number, it is estimated to be 60,000-61,000 from hospital deliveries in the whole year 2010.

Data collection: for all the hospital deliveries, each birth should have a case record form to be collected through regional perinatal-neonatal information network system, and assisted by co-investigators and task force group members from most level II and III hospital obstetric and pediatric departments and outreaching local (county and district) level I hospitals and clinic services.

Study variables: maternal, fetal and neonatal biological and clinical pathological variables on prenatal, intrapartum, and postnatal examinations, interventions and outcome.

The study protocol was approved by the Ethics Committee and Scientific Committee of the Children’s Hospital of Fudan University, and adopted by all the participating hospitals through local scientific committee approval according to Chinese regulation for clinical investigation. As data were collected from observational parameters and no specific intervention was used in the protocol, informed consent was waived.

### Definitions of vital statistics and perinatal morbidities

Live birth, fetal death or stillbirth, and death during delivery are defined according to the 10^th^ revision of the international classification of diseases [15, 16], and details of birth related definitions such as GA, BW and perinatal and neonatal death rate are described elsewhere [14]. The perinatal period commences at 22 complete weeks (154 days) of GA, ended at 7 postnatal days and the neonatal period is the first 28 complete days after birth. Fetal death is synonymous of stillbirth. The fetal status, perinatal morbidities, birth defects (BD) or congenital anomalies, and major neonatal diseases are defined according to Fanaroff and Martin [17] and domestic clinical criteria. The same applies for diagnosis of pregnancy complications as obstetric pathologies of each pregnancy based on both domestic criteria adopting internationally recognized definitions [16], and presented as incidence rates using numbers of total births as denominator. Incidence of specific neonatal disease is expressed by using either total live births as denominator or total hospitalized numbers as constitutive rate where appropriate [14].

### Quality control

To ensure that all the records to be accurate and complete, on-site physician/investigator and coordinators cross checked all the variables and values, along with visiting, telephone or e-mail communication, for verification and correction of the data. General quality control inspections were also provided for birth registration or medical records from all the municipal and county hospitals, and 20% township hospitals [14]. Educational sessions were provided to ensure clinical diagnosis and management conforming to the best available domestic criteria to each level of clinical service and capacity.

### Statistical analysis

The EPIDATA database was used for datasheet recordings and statistical analysis was performed using SPSS software (v. 16.0, SPSS Inc. Chicago, IL). Numerical data were presented by the mean (standard deviation, SD) or median (interquartile range, IQR) where appropriate, using one way analysis of variance for comparisons of continuous variables between subset data. Categorical variables were represented as frequencies or rates, using Chi square test for comparison of differences. A p value <0.05 was considered statistically significant. Missing data for each variable in the analysis ranged in 0.2-0.8% and were considered acceptable. For assessment of relative risks of perinatal mortality associated with pregnancy complications, perinatal morbidities and neonatal status at birth, uni- and multi-variate logistic regression analysis was performed for clinical variables. Results are reported as odds ratio (OR) or relative risk and 95% confidence interval (CI).

## Results

### General conditions of total births and live births

Table 1 illustrates that all birth related maternal-fetal and neonatal data, as well as birth delivery related morbidities in three levels of the hospitals, and that 85% deliveries were from level I and II hospitals. It also demonstrates that more preterm birth with low BW, pregnancy complications, congenital anomalies, Apgar score ≤3 at 1-min and 5-min, high risk newborns, and neonatal deaths at delivery were seen at level II and III hospitals, suggesting centralized management of high risk pregnancy and delivery in the region.

Over the 12-month study period, there were totally 61,227 birth registries, with 99.6% (60,986) as live birth in which 60,615 had birth information collected (99% of the total births). The total birth rate was 11.3‰ (referenced to 5,400,000). There were 32,406 (53.8%) males and 27,874 females in the total births, or a male-to-female ratio of 116:100. This ratio was even higher with increasing BW strata or order of the births [14]. The information of GA and BW related general and stratified data and analysis are reported elsewhere [14]. Incidence of BD was found in 0.67% (411/60,986) including 88 (21.4%) cleft lip and palate, 53 (12.9%) finger or toe malformations, 47 (11.4%) congenital heart disease (CHD), 35 (8.5%) neural tube defects, 31 (7.5%) urogenital system malformations, 25 (6.1%) external ear malformations, 19 (4.6%) hydrocephalus, 19 (4.6%) alimentary system malformations, among others. The BD rate decreased with increasing BW, male being 0.73% and female 0.62% (p = 0.114), respectively.

### Pregnancy complications

Overall 10% (6,051/60,445) deliveries had significantly pregnancy complications as major risk factors such as premature rupture of membrane (3,290, 5.4%), hypertension (1,016, 1.7%), anemia (746, 1.2%), preeclampsia (277, 0.4%), placenta previa (181), hepatitis (174), infection (107), diabetes (85), cardiac diseases (82), placenta abruption (80), renal diseases (16), among others. Table 2 shows, from all the deliveries, maternal and perinatal status with and without major pregnancy complications. Prevalence of Cesarean (C)-section, proportions of fetal death/stillbirth, preterm birth, low birth weight, multiple births, congenital anomalies (BD), death at delivery, and neonatal death were significantly higher in those with pregnancy complications than those without. Tables 3 and 4 illustrate neonatal BW and GA strata associated pregnancy complications, birth related death rate, gender, neonatal morbidity and mortality. With increasing BW, more males and C-section deliveries were getting higher whereas preterm births, multiple births, congenital anomalies, pregnancy complications, hospitalized infants, oxygen therapy, mechanical ventilation, corticosteroid use, and major morbidities were getting lower. The same trend was true with increasing GA.

**Table 2:** **Pregnancy complication related maternal and fetal birth status**

Pregnancy complications	Yes	No	P values
All pregnancies	5,938 (10.0)	53,925 (90.0)	
Maternal age (years)	26.7±5.6	25.8±5.1	<0.001
<20	136 (2.3)	1,348 (2.5)	
20-24	2,483 (42.0)	26,168 (48.8)	
25-29	1,872 (31.7)	15,566 (29.0)	
30-34	712 (12.0)	6,228 (11.6)	
>35	706 (11.9)	4,296 (8.0)	
First delivery	4,093 (68.0)	34,921 (64.7)	<0.001
Cesarean section	4,315 (71.6)	27,553 (50.8)	<0.001
Fetal deaths/stillbirths	34 (0.6)	155 (0.3)	<0.001
Birth numbers	6,051 (10.0)	54,394 (90.0)	
Live births	6,017 (99.4)	54,239 (99.7)	
Preterm births	860 (14.2)	1,370 (2.5)	<0.001
32-36 weeks	759 (12.6)	1,174 (2.2)	
28-31 weeks	96 (1.6)	170 (0.3)	
24-27 weeks	5	26	
Post-term births	90 (1.5)	1602 (3.0)	<0.001
Low birthweight	541 (9.0)	1140 (2.1)	<0.001
Multiple-births	224 (3.7)	859 (1.6)	<0.001
Congenital anomalies	56 (0.9)	257 (0.5)	<0.001
Deaths^a^	54 (0.9)	194 (0.4)	<0.001

All values are given in numbers and percentage (% of all pregnancies or birth numbers) or means±SD. ^a^Including fetal deaths/stillbirth and neonatal deaths immediately at delivery but counted as live births.

**Table 3:** **Perinatal status and pregnancy complications in neonatal birthweight strata**

Birthweight (g)	<1,500	1,500-2,499	2,500-3,999	≥4,000	P values
Birth number (%)	154 (0.3)	1,537 (2.5)	50,450 (83.6)	8,231 (13.6)	
Males	73 (48.0)	762 (49.9)	26,133 (52.0)	5,343 (65.3)	<0.001
Gestational age (weeks)	30.1±2.9	35.9±2.9	39.7±1.2	40.2±1.0	<0.001
Birthweight (grams)	1,157±238	2,140±269	3,365±333	4,192±250	<0.001
Preterm births	152 (98.7)	950 (62.3)	1,103 (2.2)	26 (0.3)	<0.001
Multi-births	28 (18.2)	323 (21.0)	729 (1.4)	23 (0.3)	<0.001
Congenital anomalies	11 (7.1)	54 (3.5)	222 (0.4)	27 (0.3)	<0.001
Cesarean section rate	43 (28.3)	748 (49.0)	25,629 (50.9)	5,438 (66.2)	<0.001
Pregnancy complications	65 (42.2)	476 (31.0)	4,769 (9.4)	710 (8.6)	<0.001
Hypertension	12 (7.8)	105 (6.8)	758 (1.5)	133 (1.6)	<0.001
PROM	25 (16.2)	236 (15.4)	2,678 (5.3)	336 (4.1)	<0.001
Anemia	3 (1.9)	40 (2.6)	590 (1.2)	110 (1.3)	<0.001
Maternal age (years)	26.6±6.3	26.3±5.9	25.7±5.1	26.7±5.3	<0.001
Delayed childbearing	22 (14.5)	192 (12.6)	3,971 (7.9)	866 (10.6)	<0.001
>9 years’ education	15 (10.9)	256 (18.7)	8,614 (18.4)	1,397 (18.3)	0.151
Amniotic fluid volume					<0.001
Normal	123 (83.7)	1,273 (86.9)	45,686 (92.5)	7,608 (94.5)	
Polyhydramnios	11 (7.5)	27 (1.8)	331 (0.7)	105 (1.3)	
Oligohydramnios	13 (8.8)	165 (11.3)	3,395 (6.9)	342 (4.2)	
Amniotic contamination					<0.001
Normal	121 (80.1)	1,259 (84.3)	43,144 (86.2)	6,841 (83.9)	
Grade I	10 (6.6)	62 (4.1)	2,616 (5.2)	533 (6.5)	
GradeII	4 (2.6)	68 (4.5)	2,273 (4.5)	391 (4.8)	
Grade III	16 (10.6)	104 (7.0)	2,022 (4.0)	388 (4.8)	
Apgar score					
1-min ≤7	107 (74.3)	449 (29.7)	1,509 (3.0)	212 (2.6)	<0.001
1-min ≤3	59 (41.0)	115 (7.6)	153 (0.3)	17 (0.2)	<0.001
5-min ≤7	91 (63.6)	209 (13.9)	209 (0.4)	27 (0.3)	<0.001
5-min ≤3	45 (31.5)	88 (5.8)	89 (0.2)	12 (0.1)	<0.001
Hospitalization	121 (78.6)	1,209 (78.7)	4,880 (9.7)	637 (7.7)	<0.001
Oxygen therapy	103 (66.9)	565 (36.8)	1,043 (2.1)	122 (1.5)	<0.001
Mechanical ventilation	67 (43.5)	246 (16.0)	255 (0.5)	23 (0.3)	<0.001
Postnatal steroids	10 (6.5)	71 (4.6)	488 (1.0)	72 (0.9)	<0.001
Surfactant therapy	18 (11.7)	57 (3.7)	14	1	<0.001
Pneumonia	88 (57.1)	910 (59.2)	2,532 (5.0)	326 (4.0)	<0.001
Asphyxia	31 (20.1)	214 (13.9)	504 (1.0)	59 (0.7)	<0.001
RDS	56 (36.4)	178 (11.6)	132 (0.3)	4	<0.001
IVH	50 (32.5)	468 (30.4)	670 (1.3)	62 (0.8)	<0.001
Hyperbilirubinemia	7 (4.5)	120 (7.8)	1448 (2.9)	202 (2.5)	<0.001
Deaths^a^	55 (35.7)	93 (6.1)	88 (0.2)	10 (0.1)	<0.001

All values are numbers and percentage (% of total births in each birthweight category) or means±SD; ^a^including fetal deaths/stillbirth and neonatal deaths immediately at delivery but counted as live births.

*Abbreviations*: *PROM* premature rupture of membrane, *RDS* respiratory distress syndrome, *IVH* intraventricular hemorrhage.

**Table 4:** **Perinatal status and pregnancy complications in neonatal gestational age strata**

Gestational age (weeks)	<32	32-36	37-41	≥42	P values
All births^a^	302 (0.5)	1,937 (3.2)	56,331 (93.5)	1,694 (2.8)	
Males	164 (54.8)	1,076 (56.0)	30,134 (53.8)	838 (49.7)	0.002
Gestational age (weeks)	29.8±1.8	35.3±1.3	39.8±1.0	42.4±0.5	<0.001
Birthweight (g)	1,727±709	2,572±562	3,475±442	3,596±443	<0.001
Low birthweight	258 (85.4)	844 (43.8)	572 (1.0)	6 (0.4)	<0.001
Multi-births	37 (12.3)	334 (17.2)	705 (1.3)	9 (0.5)	<0.001
Congenital anomalies	25 (8.4)	38 (2.0)	243 (0.4)	7 (0.4)	<0.001
Cesarean section	73 (24.6)	960 (49.8)	29,697 (52.8)	1,082 (64.0)	<0.001
Pregnancy complications	101 (34.0)	759 (39.3)	5,086 (9.1)	90 (5.3)	<0.001
Hypertension	12 (4.0)	127 (6.6)	855 (1.5)	17 (1.0)	<0.001
PROM	54 (17.9)	427 (22.0)	2761 (4.9)	40 (2.4)	<0.001
Anemia	4 (1.3)	60 (3.1)	667 (1.2)	14 (0.8)	<0.001
Maternal age (years)	26.9±6.3	26.7±5.9	25.8±5.1	25.1±5.0	<0.001
Delayed childbearing	45 (15.0)	262 (13.6)	4,601 (8.2)	125 (7.4)	<0.001
>9 years’ education	41 (15.4)	364 (21.4)	9,732 (18.6)	171 (10.8)	<0.001
Amniotic fluid volume					<0.001
Normal	242 (84.6)	1,651 (90.1)	51,189 (92.8)	1,484 (89.3)	
Polyhydramnios	17 (5.9)	35 (1.9)	410 (0.7)	10 (0.6)	
Oligohydramnios	27 (9.4)	147 (8.0)	3,561 (6.5)	168 (10.1)	
Amniotic contamination					<0.001
Normal	239 (81.3)	1,679 (89.3)	48,041 (86.0)	1,295 (77.1)	
Grade I	15 (5.1)	70 (3.7)	2,986 (5.3)	140 (8.3)	
Grade II	5 (5.1)	56 (3.0)	2,543 (4.6)	121 (7.2)	
Grade III	25 (8.5)	75 (4.0)	2,305 (4.1)	124 (7.4)	
1-min Apgar ≤7	187 (64.5)	463 (24.3)	1,561 (2.8)	62 (3.7)	<0.001
1-min Apgar ≤3	98 (33.8)	99 (5.2)	143 (0.3)	5 (0.3)	<0.001
5-min Apgar ≤7	152 (52.4)	179 (9.4)	198 (0.4)	8 (0.5)	<0.001
5-min Apgar ≤3	80 (27.6)	75 (3.9)	72 (0.1)	4 (0.2)	<0.001
Hospitalized rate	208 (68.9)	1,445 (74.6)	4,961 (8.8)	123 (7.3)	<0.001
Oxygen therapy	156 (51.7)	656 (33.9)	966 (1.7)	36 (2.1)	<0.001
Mechanical ventilation	111 (36.8)	253 (13.1)	206 (0.4)	9 (0.5)	<0.001
Surfactant therapy	33 (10.9)	49 (2.5)	8 (0)	0 (0)	<0.001
Pneumonia	167 (55.3)	1,064 (54.9)	2,494 (4.4)	69 (4.1)	<0.001
Asphyxia	59 (19.5)	232 (12.0)	493 (0.9)	16 (0.9)	<0.001
RDS	92 (30.5)	183 (9.4)	90 (0.2)	0 (0)	<0.001
Deaths^b^	93 (30.9)	76 (4.0)	73 (0.1)	4 (0.2)	<0.001

Values are given in numbers and percentage (% of all births in each gestational age category) or mean±SD. ^a^Percentage of total numbers of all births, including fetal death/stillbirths and neonatal death at delivery (see Table 1); ^b^including fetal deaths/stillbirth and neonatal deaths immediately at delivery but counted as live births.

*Abbreviations:* *PROM* premature rupture of membrane; Apgar, Apgar score of life signs; RDS, respiratory distress syndrome.

Table 5 demonstrates mode of delivery related perinatal status and complications. In those by vaginal delivery, there was 0.7% as dystocia requiring assisted operational procedures. There was difference of C-section rate between those whose BW was <2,500 or ≥2500 g (47.1% vs. 53.1%, P < 0.001). The C-section rate for those with pregnancy complications was significantly higher (Table 2). More neonates from C-section had history of abnormal amniotic fluid quantity, CHD and respiratory distress syndrome, required oxygen therapy and mechanical ventilation in contrast to fewer jaundice and cephalohematoma than those from vaginal delivery. From Table 6, more pregnancy complications, preterm births and low BW rates, and congenital anomalies (BD) were seen in those whose maternal age >35 years old. In those whose age <20 years old, there were also high proportions of preterm births and low BW rates. The average maternal age was 25.9 ± 5.1 (median 24, range 16–49) years old, and 2.47% (1,490/60,209) were <20 years old. Delayed child-bearing (≥35 y) accounted for 8.4% (5,065/60,209). Table 7 shows high risk pregnancy and perinatal morbidities in association with maternal school education years, where 1.5% were illiteracy.

**Table 5:** **Mode of delivery related perinatal status, complications and neonatal morbidities**

Mode of delivery	Vaginal ^b^	Cesarean section	P values
All births^a^	28,481 (47.1)	31,964 (52.9)	
Males	14,683 (51.8)	17,640 (55.4)	<0.001
Gestational age (weeks)	39.6±1.6	39.7±1.4	<0.001
Birthweight (grams)	3,387±475	3,488±500	<0.001
Preterm births	1,191 (4.2)	1,033 (3.2)	<0.001
Low birthweight	887 (3.1)	791 (2.5)	<0.001
Multi-births	242 (0.9)	844 (2.6)	<0.001
Congenital anomalies	142 (0.5)	171 (0.5)	0.519
Pregnancy complications	1,715 (6.0)	4,315 (13.5)	<0.001
Hypertension	215 (0.8)	797 (2.5)	<0.001
PROM	1,045 (3.7)	2,234 (7.0)	<0.001
Anemia	273 (1.0)	470 (1.5)	<0.001
Maternal age (years)	25.5±5.0	26.2±5.2	<0.001
Delayed childbearing	2,110 (7.5)	2,948 (9.3)	<0.001
>9 years’ education	4,451 (16.8)	5,851 (19.8)	<0.001
Amniotic fluid volume			<0.001
Normal	26,999 (96.5)	27,774 (89.1)	
Polyhydramnios	150 (0.5)	325 (1.0)	
Oligohydramnios	837 (3.0)	3,072 (9.9)	
Amniotic contamination			<0.001
Normal	24,569 (86.6)	26,877 (85.1)	
Grade I	1,476 (5.2)	1,753 (5.6)	
Grade II	1,146 (4.0)	1,594 (5.0)	
Grade III	1,178 (4.2)	1,354 (4.3)	
Apgar score			
1-min ≤7	1,253 (4.4)	1,024 (3.2)	<0.001
1-min ≤3	256 (0.9)	88 (0.3)	<0.001
5-min ≤7	367 (1.3)	170 (0.5)	<0.001
5-min ≤3	189 (0.7)	42 (0.1)	<0.001
Hospitalization	3,137 (11.0)	3,715 (11.6)	0.019
Oxygen therapy	777 (2.7)	1,056 (3.3)	<0.001
Mechanical ventilation	205 (0.7)	387 (1.2)	<0.001
Postnatal corticosteroids	284 (1.0)	358 (1.1)	0.076
Surfactant use	34 (0.1)	56 (0.2)	0.047
Pneumonia	1,779 (6.2)	2,080 (6.5)	0.098
Jaundice^c^	892 (3.1)	887 (2.8)	0.010
Asphyxia	357 (1.3)	451 (1.4)	0.092
Intraventricular hemorrhage	593 (2.1)	655 (2.1)	0.776
Hypoxic-ischemic encephalopathy	557 (2.0)	711 (2.2)	0.021
Sepsis	736 (2.6)	815 (2.5)	0.789
Congenital heart disease	183 (0.6)	340 (1.1)	<0.001
Respiratory distress syndrome	123 (0.4)	248 (0.8)	<0.001
Cephalohematoma	216 (0.8)	39 (0.1)	<0.001
ABO hemolysis	105 (0.4)	108 (0.3)	0.285
Deaths^d^	202 (0.7)	42 (0.1)	<0.001

Values are given in numbers and percentage (% of all births in each delivery category) or mean±SD. ^a^Percentage of total numbers of all births, including fetal death/stillbirths and neonatal death at delivery (see Table 1); ^b^including 397 with assisted operation procedures; ^c^including hyperbilirubinemia; ^d^including fetal deaths/stillbirth and neonatal deaths immediately at delivery but counted as live births.

*Abbreviations*: *PROM* premature rupture of membrane, *Apgar* Apgar score of life signs.

**Table 6:** **Maternal age related perinatal status and complications**

Maternal age, years	<20	20-34	≥35	P values
Live births, n (%)^a^	1,490 (2.5)	53,654 (89.1)	5,065 (8.4)	
Males	761 (51.2)	28,667 (53.7)	2,763 (54.8)	0.054
Gestational age (weeks)	39.7±1.7	39.7±1.5	39.4±1.7	<0.001
Birthweight (grams)	3,329±478	3,442±485	3,465±552	<0.001
Preterm birth rate	74 (5.0)	1,847 (3.5)	307 (6.1)	<0.001
Low birthweight	56 (3.8)	1,406 (2.6)	214 (4.2)	<0.001
Multi-births	18 (1.2)	939 (1.8)	121 (2.4)	0.001
Congenital anomalies	5 (0.3)	273 (0.5)	33 (0.7)	0.245
Cesarean section rate	672 (45.1)	28,141 (52.6)	2,948 (58.3)	<0.001
Pregnancy complications	138 (9.3)	5,162 (9.6)	717 (14.2)	<0.001
Hypertension	15 (1.0)	795 (1.5)	200 (3.9)	<0.001
PROM	75 (5.0)	2,920 (5.4)	280 (5.5)	0.757
Anemia	17 (1.1)	637 (1.2)	89 (1.8)	0.002
Maternal age (years)	18.6±0.6	24.9±3.6	38.0±2.5	<0.001
>9 years’ education	62 (4.4)	9,827 (19.7)	401 (8.5)	<0.001
Amniotic fluid volume				0.005
Normal	1,344 (92.0)	48,608 (92.6)	4,568 (92.3)	
Polyhydramnios	16 (1.1)	396 (0.7)	61 (1.2)	
Oligohydramnios	101 (6.9)	3,490 (6.6)	318 (6.4)	
Amniotic contamination				<0.001
Normal	1,245 (84.1)	45,731 (86.0)	4,245 (84.6)	
Grade I	93 (6.3)	2,843 (5.3)	267 (5.3)	
Grade II	84 (5.7)	2,412 (4.5)	234 (4.7)	
Grade III	58 (3.9)	2,196 (4.1)	269 (5.4)	
Apgar score				
1-min ≤7	69 (4.6)	1,961 (3.7)	249 (4.9)	<0.001
1-min ≤3	14 (0.9)	280 (0.5)	52 (1.0)	<0.001
5-min ≤7	22 (1.5)	443 (0.8)	73 (1.5)	<0.001
5-min ≤3	13 (0.9)	181 (0.3)	40 (0.8)	<0.001
Deaths^b^	10 (0.7)	203 (0.4)	34 (0.7)	0.002

Values are given in numbers and percentage (% of live births in each maternal age category) or mean±SD; ^a^Percentage of the total number of all live birth; ^b^including fetal deaths/stillbirth and neonatal deaths immediately at delivery but counted as live births.

*Abbreviations*: *PROM* premature rupture of membrane.

**Table 7:** **Maternal education related perinatal status and complications**

Education years	>12	10-12	7-9	<7	P values
All births^a^	4,396 (7.8)	5,943 (10.6)	39,702 (70.5)	6,249 (11.1)	
Males	2,319 (53.4)	3,056 (51.9)	21,318 (53.9)	3,388 (54.4)	0.017
Gestational age (weeks)	39.5±1.4	39.6±1.5	39.7±1.5	39.6±1.7	<0.001
Birthweight (g)	3,462±472	3,447±485	3,446±485	3,402±524	<0.001
Preterm birth rate	157 (3.6)	248 (4.2)	1,254 (3.2)	307 (5.0)	<0.001
Low birthweight	108 (2.5)	163 (2.8)	996 (2.5)	240 (3.8)	<0.001
Multi-births	86 (2.0)	93 (1.6)	673 (1.7)	130 (2.1)	0.073
Congenital anomalies	21 (0.5)	34 (0.6)	190 (0.5)	47 (0.8)	0.041
Cesarean section	2,609 (59.6)	3,242 (54.7)	20,657 (52.1)	3,093 (49.6)	<0.001
Pregnancy complications	634 (14.6)	715 (12.1)	3,310 (8.4)	616 (9.9)	<0.001
Pregnant hypertension	72 (1.6)	104 (1.7)	594 (1.5)	157 (2.5)	<0.001
PROM	432 (9.8)	427 (7.2)	1,827 (4.6)	258 (4.1)	<0.001
Pregnant anemia	44 (1.0)	92 (1.5)	420 (1.1)	93 (1.5)	<0.001
Maternal age (years)	26.8±3.0	25.5±4.3	25.3±4.9	28.9±7.0	<0.001
Delayed childbearing	106 (2.4)	295 (5.0)	2,713 (6.9)	1,590 (25.5)	<0.001
Amniotic fluid volume					0.259
Normal	3,856 (92.1)	5,395 (93.5)	36,223 (92.9)	5,665 (92.6)	
Polyhydramnios	31 (0.7)	34 (0.6)	283 (0.7)	47 (0.8)	
Oligohydramnios	298 (7.1)	344 (6.0)	2,492 (6.4)	406 (6.6)	
Amniotic contamination					<0.001
Normal	3,750 (87.5)	5,083 (86.7)	33,942 (86.1)	5,283 (85.1)	
Grade I	170 (4.0)	253 (4.3)	2,154 (5.5)	372 (6.0)	
Grade II	150 (3.5)	247 (4.2)	1,816 (4.6)	303 (4.9)	
Grade III	218 (5.1)	283 (4.8)	1,518 (3.8)	250 (4.0)	
1-min Apgar ≤7	219 (5.0)	195 (3.3)	1,413 (3.6)	269 (4.3)	<0.001
1-min Apgar ≤3	16 (0.4)	26 (0.4)	207 (0.5)	68 (1.1)	<0.001
5-min Apgar ≤7	44 (1.0)	37 (0.6)	308 (0.8)	92 (1.5)	<0.001
5-min Apgar ≤3	8 (0.2)	16 (0.3)	145 (0.4)	49 (0.8)	<0.001
Deaths^b^	8 (0.2)	17 (0.3)	155 (0.4)	55 (0.9)	<0.001

Values are given in numbers and percentage (% of all births in each maternal education year category) or mean±SD. ^a^Percentage of total numbers of all births, including fetal death/stillbirths and neonatal death at delivery (see Table 1); ^b^including fetal deaths/stillbirth and neonatal deaths immediately at delivery but counted as live births.

*Abbreviations*: *PROM* premature rupture of membrane, *Apgar* Apgar score of life signs.

### Perinatal complications and hospitalized neonatal patients

There were 6,872 neonates admitted to the ward including 4,332 male and 2,540 female (male-to-female ratio 1.71:1), in which 43%, 26.2%, 11.1% and 19.8% were admitted on day 1, 2–7, 8–14, 15–28, respectively, with 75% of the total as rural origin. In 18.8% of the hospitalized neonatal patients, their mother had pregnancy complications [14], and these incidences declined with increasing BW. For the pregnancy complications before delivery in the mothers of all hospitalized neonates, 547 (8.0%) had premature rupture of the membrane, 248 (3.6%) fever and infection, 160 (2.3%) hypertension, 105 (1.5%) preeclampsia, 77 (1.1%) placenta previa, 40 (0.6%) anemia or placenta abruption each, 29 (0.4%) diabetes, 27 (0.4%) liver diseases, 26 (0.4%) hematological diseases, 17 (0.2%) syphyllis, 14 (0.2%) cardiac diseases, 7 systemic lupus erythematosus, 5 renal diseases, 4 epilepsy, 4 hyperthyroidism, 3 hypothyroidism, etc. In the hospitalized and critically ill neonatal patients, major diagnoses were severe sepsis and septic shock, diaphragmatic hernia, disseminated intravascular coagulation, pulmonary hemorrhage, cardiac failure, respiratory distress syndrome, pneumothorax, necrotizing enterocolitis, each with a mortality of 78.6%, 60%, 60%, 53.8%, 38.9%, 18.9%, 17.0% and 9.1%, respectively. In all these patients, 26.8% received oxygen therapy, 8.6% were mechanically ventilated, 99.2% treated with antibiotics, 9.3% corticosteroids, and 1.3% surfactant therapy, whereas 34.9% recovered, 56.2% improved, 4.8% requested own discharge, 2.4% were given up or died, 1.7% transferred to other hospitals. Average length of hospital stay was 8.5 ± 6.3 days, and average cost of hospital stay was 4,182 ± 4,033 Yuan (CNY, 6.5 = 1 USD in 2010).

### Uni- and multi-variate logistic regression analysis of risks for perinatal mortality

Table 8 illustrates the results from uni- (part A) and multi-variate (part B) binary logistic regression analysis of risks of perinatal mortality from clinical variables associated with pregnancy complications, perinatal morbidities and neonatal status. In both uni- and multi-variate logistic regression analysis, BD, low GA and BW are the three most significant contributing factors, indirectly reflecting the influence of pregnancy complications and other perinatal risks, whereas gender was not a contributing factor.

**Table 8:** **Uni- and multi-variate regression analysis of risk factors for perinatal mortality**

A. Uni-variate regression analysis for perinatal mortality					
Variables	Category	Mortality (%)*	OR	95% CI	P values
Gender	Male	0.4	1.154	0.898-1.481	0.262
	Female	0.4			
Multi-births	No	0.4	3.984	2.426-6.543	<0.001
	Yes	1.5			
Congenital anomalies	No	0.3	74.260	54.067-101.996	<0.001
	Yes	19.2			
Pregnancy complications	No	0.4	2.496	1.844-3.377	<0.001
	Yes	0.9			
Birthweight (grams)	≥2500	0.2	57.700	44.478-74.852	<0.001
	<2500	8.8			
Gestational age (weeks)	≥37	0.1	61.782	47.019-81.180	<0.001
	<37	7.6			
Maternal education (years)	≥10	0.2	1.888	1.247-2.860	0.002
	<10	0.5			
Maternal age (years)	<35	0.4	1.743	1.212-2.506	0.002
	≥35	0.7			
Amniotic contamination	No	0.2	8.374	6.454-10.864	<0.001
	Yes	1.6			
Amniotic fluid volume	Normal	0.3	4.894	3.661-6.542	<0.001
	Abnormal	1.5			
**B. Multi-variate regression analysis for perinatal mortality**					
			**95% CI**		
**Variables**	Reference**	**OR**	**Lower**	**Upper**	**P values**
Gender	Male	1.240	0.896	1.715	0.194
Multi-births	No	2.396	1.196	4.799	0.014
Congenital anomalies	No	35.200	20.304	61.026	<0.001
Pregnancy complications	No	1.900	1.215	2.969	0.005
Birthweight (grams)	≥2500	5.397	3.458	8.442	<0.001
Gestational age (weeks)	≥37	32.305	20.463	50.998	<0.001
Education years	≥10	1.789	1.058	3.027	0.030
Maternal age (years)	<35	1.133	0.694	1.847	0.618
Amniotic contamination	No	10.995	7.824	15.452	<0.001
Amniotic fluid volume	Normal	2.354	1.583	3.502	<0.001

*Mortality denotes those numbers of fetal deaths, stillbirths and early neonatal deaths in each category based on the total numbers of deaths listed in Table 1, 2, 3, 4, 5, 6 and 7.

**As references of the opposite category of corresponding variables in this table part A.

*Abbreviations*: *OR* odds ratio, *CI* confidence interval of OR.

## Discussions

This study estimated impact of overall high risk pregnancy and delivery at three levels of hospitals based on data file from complete birth population, stratified GA and BW of the neonates, and maternal mode of delivery, age and education, confirmed by uni- and multivariate logistic regression analyses of the risks of perinatal deaths. These results are evidences linking the major pregnancy complications to perinatal and neonatal morbidity and mortality, accounting in part for all the hospitalized neonates, and should be regarded as benchmark in this study field. These results should also be complementary to our previous report on perinatal and neonatal mortalities, premature birth rate, and consist of a detailed description of the perinatal care and delivery in a regional Chinese perinatal care system [14], and to the understanding of previous reports on the trend of neonatal mortality in China in comparison with the worldwide development [1–8]. The 151 level I-III hospitals in this region served for >60,000 annual deliveries from 5.4 millions of population, and their capacity and quality were very representative and close to average of the emerging regions in east and mid-land provinces of the country.

There was a clear trend of high risk pregnancy and delivery among three levels of hospitals in this region as reflected by differences of BD, preterm births, multiple births, congenital anomalies, pregnancy complications, and stress of fetus and newborns at delivery. It is possible that there was limitation at low level hospitals in distinguishing these situations in daily practice, however, this was the first time reported as fetal death and stillbirth rate from Chinese regional complete data files of regional birth registry. There was a clear trend that higher pregnancy complications and fetal deaths (including stillbirth) and early neonatal death were found mainly in the level II and III hospital deliveries (Table 1). The mothers in the level III hospitals had 2–4 times as high the pregnancy complications contributing to fetal and neonatal adverse outcome and morbidity as those in the level I hospitals (Table 1). The 10% pregnancy complications in the whole births of this survey is similar to 11% in a previous, small scale survey of 5,822 live births in 2006–2007 in Julu county hospitals, of approximate 400,000 population, Hebei province, one of the coastal regions in China [18]. The specific pregnancy complications in this survey, such as pregnant associated hypertention and preeclampsia, anemia, placenta previa, diabetes and others, were lower than that in the international and domestic levels [19–24]. As 85% of the total deliveries were at level I and II hospitals with variable, but substantial, incidence of pregnancy complications, challenge exists as to inappropriate diagnosis and underreporting, and to what magnitude the overall perinatal risks may be reduced in association with multiple factors such as facilities, caregiver competence, service standard, health insurance, and socioeconomic and socio-cultural aspects, etc.

There is possibility that incidence of pregnancy complications in all the deliveries from the level I hospitals may be underreported even if the medical care for the complications was available there. Mechanisms underlying such situation were due to insufficient clinically diagnostic experience and management or inappropriate response for concerns of liability from more reporting of the pregnancy complications. Nevertheless, this would be common in regions where perinatal care system is not properly organized, and criteria for diagnosis and management of major pregnancy morbidities and complications were not uniformly executed [7, 8, 10, 13]. These imply bias due to inappropriate diagnosis and incomplete data collection from some lower level hospitals, hence is direction of improvement in local infrastructure of perinatal and medical care for those with pregnancy complications, and information reporting system. Although in some well developed prefectural regions of the coastal provinces (such as in Zhejiang), delivery at level I hospital is no longer eligible in recent years, the delivery at level I hospital should remain for substantial proportion (20-40%) of rural residents, especially in regions of low economic development and poor perinatal care conditions [11, 12].

Mode of delivery was apparently associated with risks due to pregnancy complications [25–27] (Table 5). Those with C-section delivery had higher rate of pregnancy complications, especially premature rupture of the membrane and hypertension whereas multiple pregnancy and abnormal amniotic fluid volume were also associated. High C-section deliveries at level I and II hospitals should have contributed considerably to the overall C-section rate, in which the non-medical indication related might be accounted for a substantial proportion, indicating it necessary to standardize routine service at lower level obstetrics. Our current data reveal that a high C-section rate above 50% was seen in those with maternal age 20–34 years, no pregnancy complications, no BD or congenital anomalies, no amniotic fluid contaminations or abnormal volume, suggesting substantial numbers of delivery by C-section had no medical indications, or as low risk pregnancy. The morbidity in the neonates hospitalized tended to be severe and complicated in C-section deliveries as more required oxygen therapy and mechanical ventilation, and more respiratory distress syndrome and CHD were found. Although those delivered at level I and II hospitals accounted for almost 2/3 of pregnancy complications in the total (Table 1), as we did not have the data on severity of pregnancy complications in these hospitals, more severe pregnancy complications should have been centered and managed in the level III hospitals. This trend should have been more prominent and relevant in the emerging regions, requiring advanced perinatal care system emphasizing on routine screening services for risk factors associated with adverse pregnancy along with a close follow-up for pregnancy women at risk of observed pregnancy complications during prenatal care to minimize risk of pregnancy complications, hence possibility of C-section delivery reduction.

The social, cultural and economic factors may have influenced the regional perinatal and neonatal outcome. This study focused on mode of delivery, maternal age and education status. Maternal age should be an overt factor contributing to perinatal risks and neonatal outcome [28–32]. In those with early (<20 years old) and delayed (≥35 y) child bearing, relatively higher preterm birth rate (5% and 6.1%, respectively) were found (Table 6), along with higher rate of BD, pregnancy complications, C-section rate, lower Apgar score at 1- and 5 min, neonatal deaths rate, mainly seen in those ≥35 y. Maternal education years also had impact on pregnancy complications and neonatal outcome [33]. We found those with education <7 years had relatively higher percentage of delayed child bearing age (i.e., having more than 2 children), preterm births, low BW, lower Apgar score at 1- and 5 min, neonatal deaths rate (Table 7). It is reported that the incidence of fetal and infant mortality were inversely related to maternal educational gradient: higher with a lower level of education, a determinant of the incidence of fetal and post-neonatal death but not of early and late neonatal death (0–27 days) [33]. Taking the results from uni- and multi-variate logistic regression analysis, pregnancy complications as well as multiple births, BD, and perinatal morbidities and mothers’ biological and social status were significantly associated with the perinatal and neonatal outcome, indirectly reflecting the influence of pregnancy complications and other perinatal risks.

For limitation and implication of the study, as discussed above, in addition to incomplete data from lower level clinics, there are several major limitations of the data presentation. First, there is a lacking of causal relationship between those of maternal complications and corresponding neonatal outcome. Secondly, there is a lacking of clues for severity of the pregnancy complications and their child delivery hospital levels. To analyze and understand these relationships, it may help to define the function and capacity of perinatal care at level II and III hospital, and standardize the care with license for maternal and neonatal care, corresponding delivery and medical facilities, potentiating enormous social health cost-effectiveness assessment and follow-up analysis in clinical economics. Thirdly, the study results do not reflect efficacy of specific measurements in the regional perinatal care system with local conditions that might have restricted more efficient infrastructure building up. For example, delivery was disseminated in 129 level I hospitals, and low Apgar score and neonatal deaths were still present to certain extent in all the births. Acquisition of adequate perinatal information tended to be difficult when we scrutinized 20% of the reported case records of all the births during the two major inspections of the survey, suggesting that the centralized deliveries at level II and III hospitals, and some upgraded level I hospitals, should be the direction of future development of the regional perinatal care system. Although the data may not be used to predict single hospital service quality, it may be used to compare different regions in health care standard and development. We consider current survey should be replicable from different regional data file to provide more reliable and comprehensive estimation of perinatal and neonatal morbidity and mortality and related high risk pregnancy, including cohort study of high risk pregnancy, delivery and neonatal outcome.

## Conclusion

In conclusion, this survey demonstrates pregnancy complications and related perinatal morbidity as risk factors impacting on neonatal outcome, associated with hospital levels, maternal status, mode of delivery and major neonatal pathologies. We anticipate that more surveys are needed using the same concept and methodology in different regions to generate more comprehensive database and explore causal relations of high risk pregnancy to neonatal care and outcome, thereby ensuring development of measures and programs for the improvement by perinatal and neonatal care givers, policy makers and administrators for woman and infant health.

## Abbreviations

BD: Birth defects
BW: Birth weight
CHD: Congenital heart disease
GA: Gestational age.
